# The Impact of Endometriosis on the Health of Women

**DOI:** 10.1155/2015/365951

**Published:** 2015-07-21

**Authors:** Claude L. Hughes, Warren G. Foster, Sanjay K. Agarwal, Liselotte Mettler

**Affiliations:** ^1^Cardiovascular & Metabolic Diseases, Quintiles, Inc., 5927 S. Miami Boulevard, Morrisville, NC 27560, USA; ^2^Department of Obstetrics and Gynecology, Duke University Medical Center, Durham, NC 27710, USA; ^3^Department of Obstetrics and Gynecology, McMaster University, Hamilton, ON, Canada L8S 4K1; ^4^Department of Obstetrics and Gynecology, University of California, San Diego, San Diego, CA 92037-1300, USA; ^5^Department of Gynecology and Obstetrics, University Medical Center Schleswig-Holstein, Campus Kiel, Arnold-Heller-Straße 3, House 24, 24105 Kiel, Germany

In this special issue, we have secured a series of insightful articles from experts in this field of research and care to address endometriosis from a broad and longitudinal perspective. Specifically, this special issue brings forward several articles with novel insights into the pathogenesis of endometriosis and addressing the state-of-the-art in clinical markers of endometriosis. We think this collection of papers will help focus attention on how the adverse health impacts of endometriosis often compromise our patients in diverse ways for years to decades of their lives. The cumulative impact of endometriosis on the health of women across the lifespan can be parsed into two sets of issues, namely, those of (1) assessment and treatment of the pelvic/intraperitoneal compartment and (2) potentially systemic more remote risks, in terms of location (other tissues/organs) and time in the lifespan. As the guest editors for this special issue, we offer a plea to you, our colleagues. Endometriosis is indeed a disease with unique pathophysiology but can we please abandon use of terms implying that it is a disease of deep “enigmatic” mystery? We know that it is a subchronic to chronic predominantly intraperitoneal, often progressive and destructive, inflammatory disease that has a massive impact on the health of many postpubertal women. Given the known role of chronic inflammation as a prominent factor in the occurrence of numerous other diseases, we must determine the extent to which this process in the intraperitoneal compartment does or does not “spill over” into systemic inflammation that creates broader health risks. It is only with a comprehensive perspective that we can develop and implement a coherent strategy for care of those who suffer from endometriosis.

“It is our view that there are ample opportunities to pursue research regarding the impact of endometriosis across the lifespan and to pursue development of a number of therapeutics that could plausibly be expected to be both safe and beneficial for women suffering with this disease. This is not an argument for taking our eye off the prize meaning endometriosis as the primary disease; rather we owe it to each of our patients to consider all of the impacts at all times in life that this disease may incur. We urge deeper and broader understanding of such risks and pursuit of safe and effective interventions for the care of our patients” [1].

*Claude L. Hughes* *Warren G. Foster* *Sanjay K. Agarwal* *Liselotte Mettler*
